# A European Début: The Asian Parasitoid *Encarsia nipponica* Targets the Invasive *Aleurocanthus spiniferus* in Northern Italy

**DOI:** 10.3390/insects16111181

**Published:** 2025-11-19

**Authors:** Elena Costi, Daniele Giannetti, Michele Cesari, Carmelo Rapisarda, Andrew Polaszek, Robert L. Kresslein, Lara Maistrello

**Affiliations:** 1 Department of Life Sciences, University of Modena and Reggio Emilia, Via G. Amendola 2, 42122 Reggio Emilia, Italy; elecosti@unimore.it (E.C.); daniele.giannetti@unipr.it (D.G.); 2 Department of Life Sciences, University of Modena and Reggio Emilia, Via G. Campi 213/d, 41125 Modena, Italy; michele.cesari@unimore.it; 3 NBFC, National Biodiversity Future Center, Piazza Marina 61, 90133 Palermo, Italy; 4 Department of Agriculture, Food and Environment, Applied Entomology Division, University of Catania, Via Santa Sofia, 100, 95123 Catania, Italy; carmelo.rapisarda@unict.it; 5 Natural History Museum, London SW7 5BD, UK; a.polaszek@nhm.ac.uk; 6 United States Department of Agriculture, Agricultural Research Service, Systematic Entomology Lab, National Museum of Natural History, Smithsonian Institution, Washington, DC 20560, USA; robert.kresslein@email.ucr.edu

**Keywords:** haplotype diversity, host–parasitoid interactions, biological control potential, Aleyrodidae, Aphelinidae, grapevine agroecosystems

## Abstract

In this study, the invasive orange spiny whitefly (“OSW”; *Aleurocanthus spiniferus*) and a species of *Encarsia* parasitising its puparia were studied in three different areas of the province of Modena (Emilia-Romagna, northern Italy): a pear orchard in Bomporto, an organic pear orchard in Carpi, and the semi-natural botanical garden “La Pica” in San Felice sul Panaro. The material of both species was collected for taxonomic and molecular studies. The abundance of OSW and parasitoid activity were surveyed in whitefly puparia, with a focus on the botanical garden. A total of 1800 leaves of *Malus domestica*, *Pyrus communis* and *Vitis vinifera* were sampled to assess whitefly infestation. The results showed a significant variation in the abundance of *A. spiniferus* puparia, with the highest infestation observed in *V. vinifera*. Morphological and molecular analyses, including mitochondrial COI and rRNA 16S for whiteflies and COI and 28S for parasitoids, confirmed the identity of *A. spiniferus* and identified the parasitoid as *Encarsia nipponica*. Molecular data also revealed the presence of three haplotypes of *A. spiniferus*, including a haplotype from China, new to Europe. The parasitisation rate by *E. nipponica* was low (0.015%). Our results provide the first European record of *E. nipponica* and new genetic data on the invasion pathways of *A. spiniferus*. Collectively, these findings offer a critical baseline for monitoring their ecological interactions and developing future pest management strategies.

## Simple Summary

In northern Italy, the orange spiny whitefly (Aleurocanthus spiniferus), an invasive insect from Asia, poses a growing threat to valuable crops. This study aimed to assess its infestation levels on different crops and to search for any natural enemies that might control it. Our research observed the highest whitefly infestation levels on grapevines, posing a risk to viticulture. Crucially, our genetic analysis also revealed a whitefly haplotype new to Europe, which was previously only known to exist in China, providing important clues about the pest’s invasion pathway. Most importantly, we report the first discovery in Europe of the Asian parasitoid wasp Encarsia nipponica, which attacks whitefly puparia. While this natural enemy is not yet widespread, its presence establishes a new parasitoid-host association in Europe. These foundational findings are highly valuable, as they provide a critical starting point for developing of environmentally friendly biological control strategies that could offer a long-term solution to protecting agriculture from this damaging pest.

## 1. Introduction

*Aleurocanthus* Quaintance & Baker (Hemiptera: Aleyrodidae) includes 93 species, predominantly found in tropical and subtropical regions [1]. Due to their widespread distribution and considerable potential threat to crops, all species within this genus are classified as quarantine pests by the European Union [2].

Among these species, *Aleurocanthus spiniferus* (Quaintance), commonly known as the orange spiny whitefly (OSW), is one of the most damaging worldwide and is particularly notable for its polyphagous habits. Native to Asia, OSW has spread to most tropical and subtropical regions, including Africa, Australia, and the Pacific Islands (Hawaii) [1,3,4,5]. More recently it has entered Europe, first reported in Italy in 2008 [6], followed by reports in Croatia (2012) [7], Montenegro (2013) [8], Greece (2016) [9], Albania (2018) [10], and France (2023) [11]. In Italy, the species was initially detected in the Lecce district of the Apulia region [6] and has since been reported from eleven regions: Basilicata, Calabria, Campania, Emilia-Romagna, Lazio, Liguria, Lombardy, Marche, Tuscany and Sicily [12,13,14,15].

In 2018 the plant protection service of Emilia-Romagna, a prominent fruit-producing region in northern Italy, especially for the cultivation of pears and grapes, detected a new pest for this region, initially identified as a species of *Aleurocanthus* [12] and subsequently as *A. spiniferus.* In the following years, more outbreaks were reported in different parts of the region. Accurate species identification within this genus is complicated by polyphenism, in which morphological traits vary depending on the host plant, requiring both taxonomic and molecular approaches for precise identification [1,2,3,4,5,6,7,8,9,10,11,12,13,14,15].

*Aleurocanthus spiniferus* infests approximately 90 host plant species, including several economically significant crops, with a notable preference for *Citrus* spp. and grapes (*Vitis vinifera* L.) [13,14,15,16]. Infestations can weaken plants by direct sap loss and indirect damage caused by honeydew production, which promotes sooty mould and alters photosynthesis [10]. As a quarantine pest, OSW also impacts international trade by hindering the export of fruits and plants, which poses a significant threat to European agriculture.

As it is a quarantine pest, the phytosanitary practices that can be applied for the containment of OSW outbreaks vary according to the areas affected (nurseries, private gardens, public green areas). Integrated pest management (IPM) strategies for the management of OSW in agricultural crops include the use of soft soaps, wetting agents, and approved synthetic insecticides, depending on regional authorisations. However, these methods often provide incomplete control and can negatively affect biocontrol agents in the agroecosystem concerned, leading to secondary pest outbreaks [17]. Several parasitoids of *A. spiniferus* have been documented worldwide [18,19], primarily within the chalcidoid family Aphelinidae. Biological control efforts have been successful globally, using parasitoids such as *Encarsia spp. including E. perplexa Huang & Polaszek [as E. opulenta (Silvestri)], E. smithi* (Silvestri), and *Amitus hesperidum* (Hymenoptera: Platygastridae) [20,21,22,23,24,25], with some cases reaching mean parasitism rates of around 80% [26]. However, due to the European “Habitats Directive” [27], the use of exotic biocontrol agents is restricted in European countries.

Recent surveys in several central and southern Italian regions (Apulia, Campania, Latium, Marche and Sicily) identify *Eretmocerus iulii* Laudonia and Melone, *E. smithi* as parasitoids of *A. spiniferus* [15,24,25,28] *Eretmocerus iulii* showed parasitisation rates ranging from 4.07% to 71.43% [15,16,17,18,19,20,21,22,23,24,25,26,27,28]. Given the threat posed by OSW, more research is needed in northern Italy to assess the impact of the pest and the role of biocontrol agents.

This study aims to confirm the identity of the *Aleurocanthus* species that occur in the Emilia-Romagna region by morphological and molecular analysis and assess the presence and role of naturally occurring parasitoids, identifying them at the species level using both morphological and molecular methods.

## 2. Materials and Methods

### 2.1. Sampled Areas

The collection of samples was carried out at three different sites in the province of Modena (Emilia-Romagna region, Italy), respectively, represented by the edge of a pear orchard located in the municipal area of Bomporto (44.73164076133763, 11.039053971141435), by an organic pear orchard in the municipality of Carpi (44.727952774732834, 10.874274022562624) and by the Botanical Garden ‘La Pica’ in San Felice sul Panaro (44.87540090470623, 11.097329719787881). The latter site is a seminatural area with the presence of agricultural and ornamental plants, covering an area of approximately 21,000 m^2^ and characterised by the presence of over 1000 plant species from all over the world.

Puparia (4th instar nymphs) of OSW were collected from all three sites (Table 1) for taxonomic studies. In the botanical garden “La Pica”, a whitefly puparia survey was also carried out in different host plant species and specimens were collected to assess levels of parasitisation. For the latter study, both *Aleurocanthus* sp. and its parasitoid samples were preserved in 100% ethanol after collection, prior to morphological and molecular identification.

### 2.2. Morphological Study of Aleurocanthus sp.

As is the accepted standard for morphological studies on whiteflies, observations were carried out on puparia, which are considered to have better documented diagnostic characters than adults. To this end, puparia of *Aleurocanthus* sp. collected from all three sampling areas were treated with cold 10% KOH until they turned light brown; they were then rinsed in 70% ethanol for 5 min, transferred to glacial acetic acid for 5 min, clove oil for 15 min and mounted in Canada balsam. The prepared slide-mounted specimens were observed using differential interference contrast (DIC) on an Olympus BX63 compound microscope (Olympus UK & Ireland, Southend-on-Sea, UK).

### 2.3. Field Survey

The survey mentioned above in the botanical garden ‘La Pica’ was carried out in July 2022 on 20 plants selected for each of the following species: *Malus domestica* (Suckow) Borkh., *Pyrus communis* L. and *Vitis vinifera* L. In each of the 60 selected plants, 30 randomly selected leaves, visual inspection was carried out on site, making a total of 600 leaves for each plant species, to quantify the level of infestation by *Aleurocanthus* sp. puparia on the three different species.

The evaluation of the parasitisation rate was carried out exclusively on 10 pear plants of the Abate fetél variety (the most common pear variety in the Emilia Romagna region), from which a total of 80 leaves were collected. Each leaf was kept in a 9 cm diameter Petri dish, and all the material was stored outdoors in the facilities of the botanical garden, protected from sunlight, under the porch of the visitor centre. Each sample/Petri dish was checked every two days for possible parasitoid emergence.

### 2.4. Data Analysis

Parasitisation impact on host populations [29], which describes the efficacy of a parasitoid in reducing a pest (parasitism level), was calculated as the number of parasitoids emerged/number of OSW pupae collected × 100.

To analyse the effect of tree species on *Aleurocanthus* sp. abundance (measured as the number of whitefly puparia per leaf), a Generalised Linear Mixed Model (GLMM) was fitted using the glmm TMB package in R 4.4.2 [30,31]. The model was specified with a negative binomial distribution to account for overdispersion in the count data. The response variable in the model was the number of *Aleurocanthus* sp. puparia per leaf, and the fixed effect included in the model was the tree species (species). The structure of the random effect included random intercepts for each plant (plant) and each leaf nested within the plant (leaf), to account for the hierarchical structure of the data, where multiple leaves were sampled from the same plant and multiple plants were sampled within each species. Before fitting the GLMM, summary statistics were calculated for the number of *Aleurocanthus* sp. puparia observed on the leaves of different tree species.

### 2.5. Morphological Study of Parasitoids

Specimens were mounted on microscope slides after Noyes [29], but without maceration in 10% KOH after DNA extraction. However, this is not a general recommendation, as in many cases proteinase K/ATL buffer extraction leaves behind rhodopsin or other eye pigments, which require further maceration in KOH. The samples were then washed in distilled water for 1 h and dehydrated for 5 min in graded ethanol of the following concentrations: 35%, 70%, 85%, 100%. After being cleared in clove oil and allowing alcohol evaporation, samples were dissected in Canada balsam. The wings, antennae, head, and remaining body parts were mounted separately on a single slide. In total, 22 females and no males, from 18 samples/Petri dishes, were examined. Different structures of the specimens were scanned with an Olympus BX63 microscope with DIC (Differential Interference Contrast) imaging. The image sections were stacked and combined using Helicon Focus software version 3.10, and the final images were edited with Adobe Photoshop 26.4.0 release.

### 2.6. Molecular Analysis

To confirm the identification of the species according to morphological characteristics, some of the collected specimens of the whitefly and its parasitoid were stored at −20 °C until they were used for molecular analysis. Genomic DNA was extracted using QuickExtract^TM^ DNA Extraction Solution (Lucigen, Middleton, WI, USA), according to the manufacturer’s protocol. As this is a non-destructive method, the samples were immediately washed with 100 μL of distilled water at the end of the extraction procedure, then replaced with 100 μL of 100% ethanol and stored as vouchers at −20 °C at Department of Life Sciences, University of Modena and Reggio Emilia. A negative extraction control was also performed with all the kit solutions but without insect tissue to check for contamination.

The genetic diversity of the examined species was analysed using mitochondrial genes cytochrome oxidase subunit I (COI) and 16S rDNA for whitefly samples and using the COI and the nuclear 28S rDNA gene for parasitoids. The markers were chosen due to their high variability and the large number of previously scored samples. In whiteflies, PCR amplification of a portion of the COI gene was carried out using primers AsFmik (5′-GTG TCC CAT TTA ATT AGT AGA GA-3′) and AsRmik (5′-GAG CCA TAA TAA AAG ACT CCA TC-3′ [32]; with the protocol described in Uesugi et al. (2016) [33]. A portion of the 16S gene was also amplified for whiteflies with the primer pair 16Sbr (5′-CCG GTC TGA ACT CAG ATC ACG T-3′) and 16 Sar (5′-CGC CTG TTT AAC AAA AAC AT-3′ [34]; with the following protocol: 35 cycles with 30 s at 94 °C, 30 s at 48 °C, and 30 s at 72 °C, with a final elongation step at 72 °C for 7 min. For parasitoids, a portion of the COI gene was amplified using primers LCO-1490 (5′-GGT CAA CAA ATC ATA AAG ATA TTG G-3′; Folmer et al. 1994) [35] and C1-N-2191 (5′-CCC GGT AAA ATT AAA ATA TAA ACT TC-3′; with a step-up procedure, with the following protocol: the initial five cycles were performed with 1 min at 94 °C, 1.5 min at 42 °C and 1.5 min at 72 °C, and they were followed by 35 cycles with 1 min at 94 °C, 1.5 min at 50 °C and 1 min at 72 °C, with a final elongation step at 72 °C for 7 min. The 28S rDNA D2 and D3 expansion regions were also amplified in parasitoids using the primer pair. D23F (5′-GAG AGT TCA AGA GTA CGT G-3′ [36]; and 28Sb (a.k.a. D3B; 5′-TCGGAAGGAACC AGC TAC TA-3′ [36], with the protocol described by Polaszek et al. [37]. The amplified products were gel-purified using the Wizard Gel and PCR cleaning (Promega, Madison, WI, USA) kits. Then both strands were subjected to sequencing reactions using the Big Dye Terminator 1.1 kit (Applied Biosystems, Foster City, CA, USA) and sequenced using an ABI Prism 3100 sequencer (Applied Biosystems, Foster City, CA, USA). Nucleotide sequences of the newly analysed samples were submitted to GenBank (accession numbers: PX434450-PX434461 for COI; PX427809-PX427810 for 16S; and PX529884-PX529889 for 28S). Chromatograms were checked for the presence of ambiguous bases and COI sequences were translated to amino acids using the invertebrate mitochondrial code implemented in MEGA11 [37,38] to check for the presence of stop codons and, therefore, pseudogenes. The nucleotide sequences were aligned with the Clustal algorithm implemented in MEGA11 (pairwise and multiple alignment parameters: Gap opening penalty: 15, Gap extend penalty: 6.66) and checked by visual inspection.

Both newly analysed and reference sequences of COI and 16S for whiteflies and COI and 28S for parasitoids (see Supplementary Material Table S1) were included in the analysis, to provide better qualitative information for possible invasion pathways. Uncorrected *p*-distances between scored haplotypes were determined using MEGA11, after checking that scored sequences were not subject to saturation using the programme DAMBE7 [39]. The relationships among haplotypes were estimated using a parsimony network by applying the method described by Templeton et al. (1992) [40], as implemented in TCS 1.21 [41] and visualised using tcsBU (https://wiki.genometracker.org/~weigang/tcsBU/, accessed on 25 September 2025) [42].

### 2.7. Phylogenetic Analysis

Six sequences of 28S D2 ribosomal DNA captured from *Encarsia nipponica* were combined with previously published sequence data from 75 outgroup taxa (Table S6). Outgroups comprised a broad sampling of species-groups of *Encarsia*, *Coccophagus* Westwood, *Coccophagoides* Girault, *Pteropterix* Westwood, *Aphytis* Howard. Sequences were aligned using the E-INS-I algorithm in MAFFT v7.490 [43]. Alignments were trimmed using spruceup with cut-off values of 0.99, 0.97, 0.95 and 0.9 [44]. Phylogenetic reconstruction was performed using maximum likelihood in IQ-TREE v. 2.2.2.6, implementing a General Time Reversible model with invariant sites and gamma-distributed rate variation (-m GTR + I + G) [45]. Ten independent iterations of maximum likelihood were run with each trimmed dataset (-runs 10). Bootstrap support was estimated from 1000 bootstrap trees constructed using ultrafast bootstrapping (-b 1000) [46].

## 3. Results

### 3.1. Morphological Study of Aleurocanthus sp.

Puparia collected in Emilia Romagna belong to the genus *Aleurocanthus* on the basis of the presence of many stout dorsal spines, of a white marginal waxy fringe and of earlier instar exuviae maintained in a stack on the dorsum. For this genus, a globally valid key for species identification based on morphological characters is currently not available. Thus, the samples collected in the present study have been identified using multiple valid keys for various local faunas, such as those provided by Martin (1999) [47], Dubey and Sundararaj (2005) [48], Dubey and Ko (2012) [49], Jansen and Porcelli (2018) [50], as well as by comparison with material stored in the CR collection, at the University of Catania, Italy.

**Figure 1:**
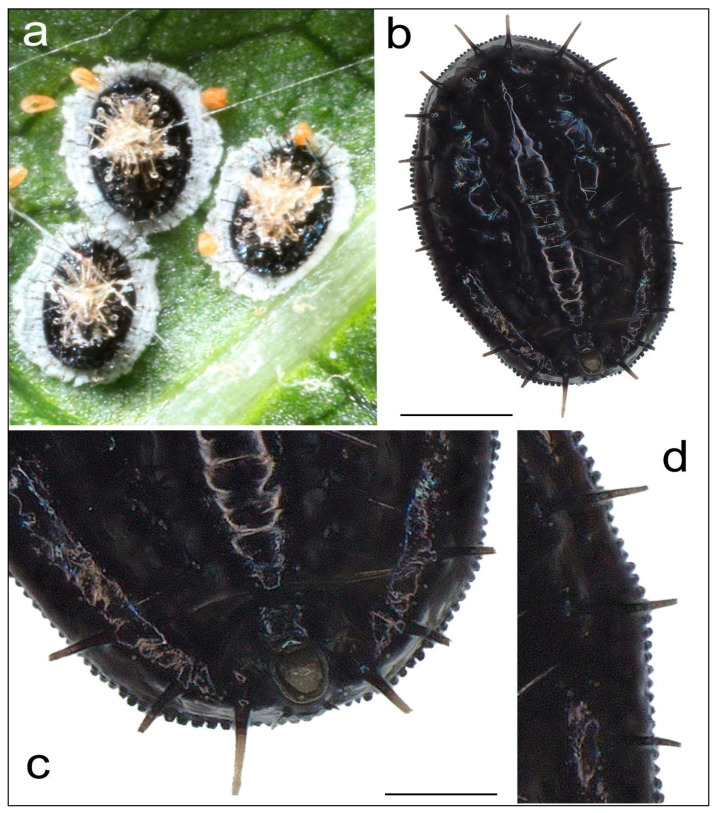
Puparia of *Aleurocanthus spiniferus* with the characteristic wax secretion (**a**), the characteristic of the dorsal spines (**b**), the posterior part of the abdomen (with the vasiform orifice) (**c**), and the marginal teeth (**d**). Scale bar: 200 µm.

The samples studied were ascribed to *Aleurocanthus spiniferus*, based on their overall shape and the width of their wax secretion (usually broad, ranging from almost 20 to 30% of puparial width) (Figure 1a,b), their dorsal spine characteristics (with the third posterior-most pair of submarginal spines always single) (Figure 1b), the overall shape of the posterior part of the abdomen (including the aspect, position and size of the vasiform orifice), with the VIII abdominal tergite around 70 μm (Figure 1c), and the shape and dimensions of marginal teeth (never less than 6 per 0.1 mm and usually exceeding 200 units in their total number) (Figure 1d).

### 3.2. Field Survey

**Figure 2:**
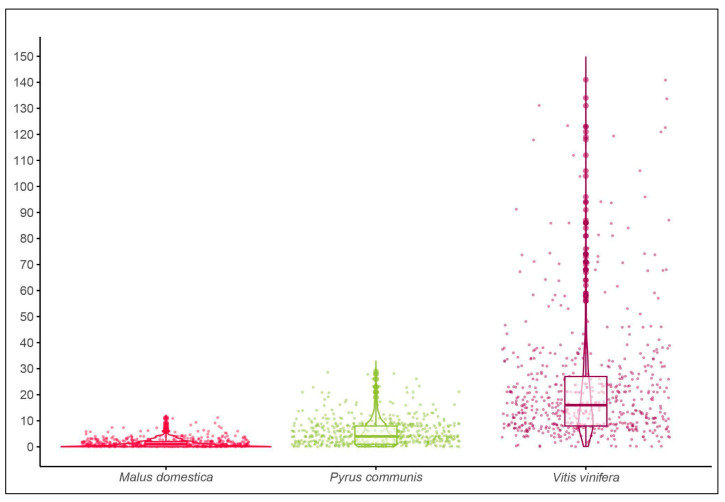
Violin plots showing the distribution of *Aleurocanthus spiniferus* puparia per leaf in host tree species: *Malus domestica*, *Pyrus communis*, and *Vitis vinifera*. Each violin plot is overlaid with a box plot and individual data points to illustrate the central tendency and spread of the data. The thick horizontal line within each box represents the median, while the box boundaries indicate the interquartile range (IQR).

(a) On the 1800 leaves collected (600 leaves per species), 2701 A. spiniferus puparia were counted. The median number [IQR-interquartile range] of A. spiniferus individuals is reported for each species (Table 2, Figure 2). Data indicate considerable variation in the abundance of A. spiniferus between the three host plant species. *Vitis vinifera* had the highest median number of individuals of *A. spiniferus* per leaf (16 [8, 28]), followed by *Pyrus communis* (4 [1, 8]), and Malus domestica (1 [0, 2]). The results of the GLMM indicate a significant effect of the host tree species on the abundance of *A. spiniferus* (Table 2, Figure 2). Specifically, the *Pyrus communis* and *Vitis vinifera* species had significantly higher numbers of *A. spiniferus* puparia compared to the reference species (Table 2). The random effects of the model showed negligible variance at the leaf level (variance = 2.77 × 10^−9^; Std. Dev. = 0.00005), suggesting that most of the variability in A. spiniferus counts was due to differences between plants (variance = 0.129; Std. Dev. = 0.36) rather than between individual leaves.
(b) Furthermore the analysis of the parasitization rate revealed 40 parasitoid samples emerged, all belonging to a single species and with a parasitoid impact on the host population of 0.015%.

### 3.3. Morphological Study of Parasitoids

**Figure 3:**
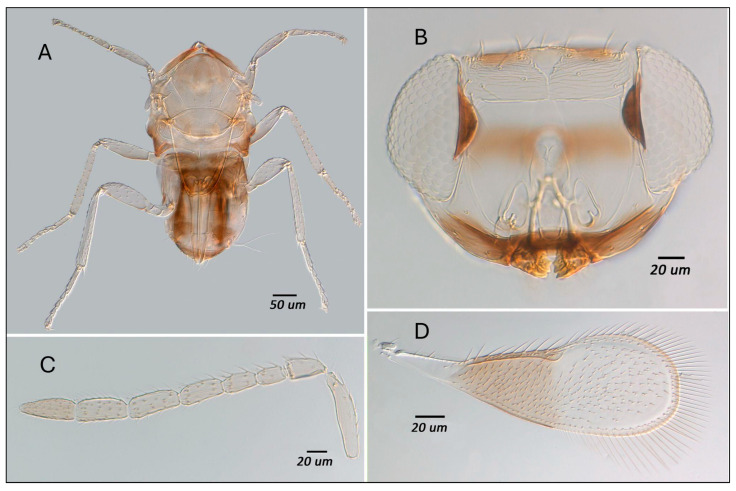
(**A**) Dorsal meso-metasoma and legs; (**B**) Face; (**C**) antenna, and (**D**) fore wing of *Encarsia nipponica***.** Photos were taken with an Olympus BX63 microscope.

Using the key to Chinese *Encarsia* by Huang and Polaszek (1998) [23], and by comparison with specimens in the collection of the Natural History Museum, London, slide-mounted specimens were identified as *Encarsia nipponica* Silvestri, 1927. The species can be diagnosed as follows: antennal formula 1,1,4,2 (Figure 3C); tarsal formula 5,5,5 (Figure 3A); fore wing with asetose area around stigmal vein and around the lower distal area; wing infuscate below marginal vein (Figure 3D); colour pattern distinctive, with the terminal antennal segment conspicuously darker than the rest of the antenna (Figure 3C); a broad dark band above the mouth opening, reaching to the lower eyes (Figure 3B); the following are dark in contrast to the rest of the body: pronotum, anterior mesoscutum, T1-T3, T5, and T7+8 of the metasoma (Figure 3A).

In Viggiani’s (1987) [51] key to Italian *Encarsia*, *E. nipponica* keys out as either *E. partenopea* (Masi) (currently a synonym of *E. inaron* (Walker)), or as *E. pergandiella* Howard, depending on the interpretation of the antenna as either having a 2-merous clava or an indistinct clava.

### 3.4. Phylogenetic Analysis

**Figure 4:**
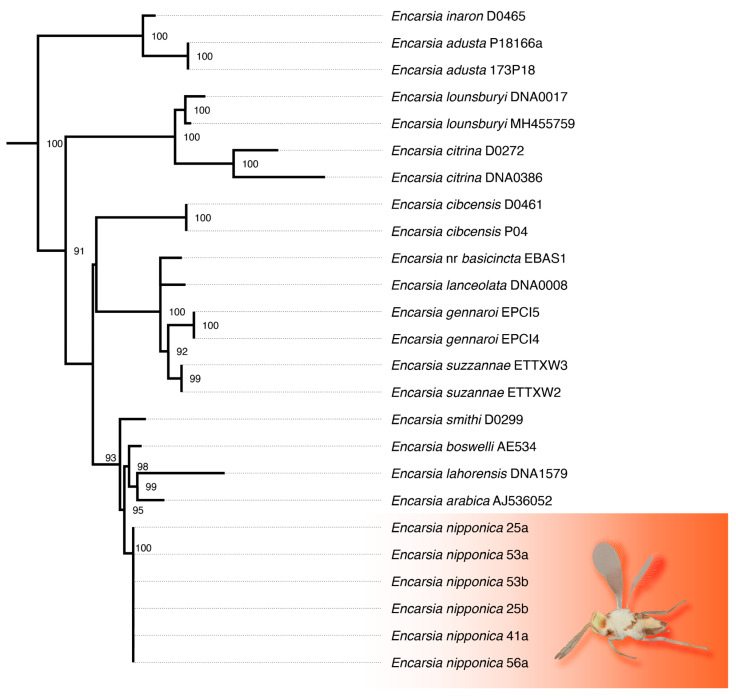
Subtree from maximum likelihood tree (IQ-TREE 2) based on 28s D2 ribosomal DNA (535 bp) from 81 taxa (6 ingroup, 75 outgroup); support values from 1000 ultrafast bootstrap replicates, shown if above 90.

Five maximum likelihood trees were constructed based on the trimmed 28S D2 rDNA alignments produced from spruceup, with the untrimmed dataset yielding the most likely overall phylogeny. While *Encarsia* was recovered as a strongly monophyletic group in all analyses, the backbone relationships therein are largely unsupported (Supplementary Material Table S2). *Encarsia nipponica* was always recovered in a strongly supported clade containing the *boswelli, citrina*, *inaron*, *lahorensis*, *longifasciata*, *parvella*, *perflava*, and *smithi* species-groups (Figure 4).

### 3.5. Molecular Analysis

**Figure 5:**
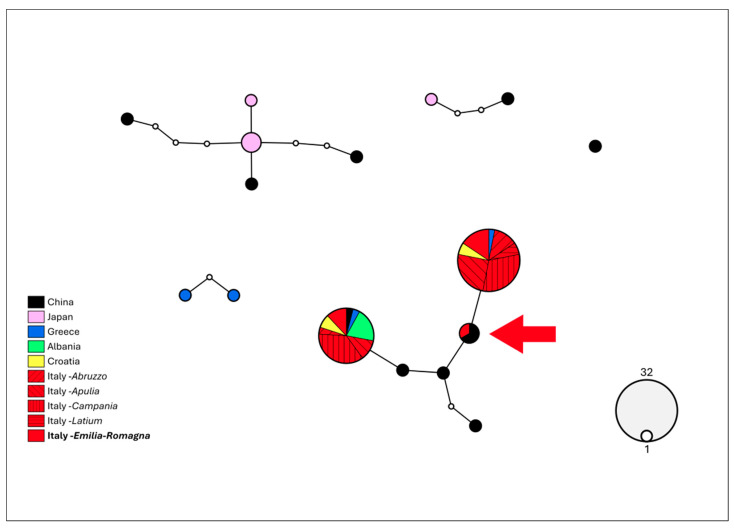
Haplotype network analysis computed on 657 bp of the COI gene of *Aleurocanthus spiniferus*. The circles denote the haplotypes, while the circle area represents the haplotype frequency. Small white circles indicate putative/missing haplotypes. Networks that fall below the value of the 95% connection limit are disconnected. The red arrow shows the new haplotype for Europe, found in Emilia-Romagna.

Uncorrected p-distances of both genes assigned Emilia-Romagna specimens to *Aleurocanthus spiniferus*, thus confirming the morphological identification, with p-distances ranging from 0–21.1% for COI and from 0 to 15.7% for 16S (Supplementary Material Tables S3 and S4). The haplotype network analysis (Figure 5) computed on the COI gene shows that the Emilia-Romagna specimens harbour three different haplotypes. Two of them had already been discovered in other Italian regions, in the Balkan region and in China, while a third one (red arrow, Figure 5), previously described for Chinese specimens, was discovered for the first time in Europe.

**Figure 6:**
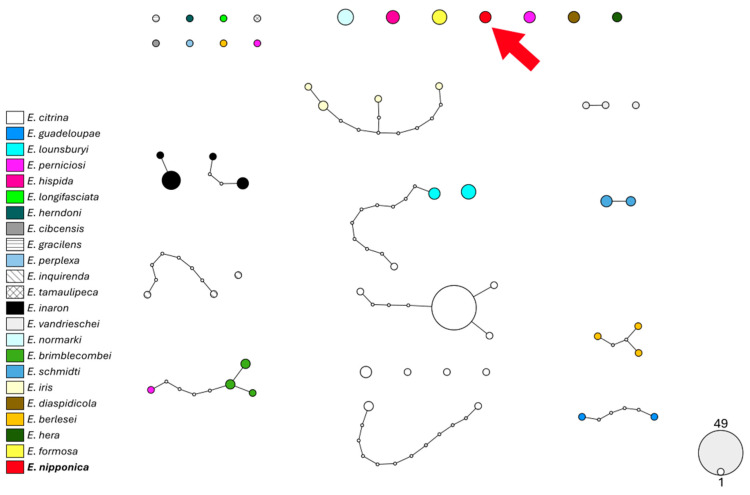
Haplotype network analysis on 672 bp of the COI gene of *Encarsia*. Circles denote haplotypes, while circle area represents haplotype frequency. Small white circles indicate putative/missing haplotypes. Networks that fall below the value of the 95% connection limit are disconnected. The red arrow shows the haplotype found in Emilia-Romagna specimens.

**Figure 7:**
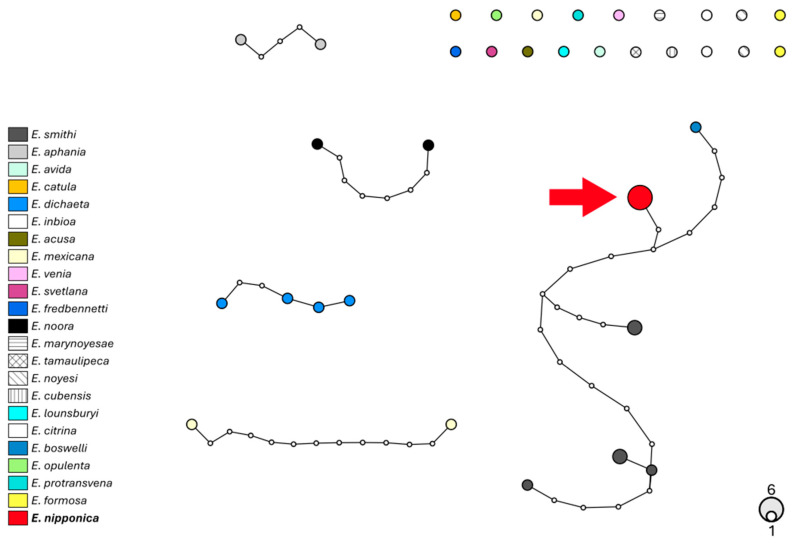
Haplotype network analysis on 858 bp of the 28S gene of *Encarsia*. Circles denote haplotypes, while the circle area represents the frequency of the haplotype. Small white circles show putative/missing haplotypes. Networks that fall below the value of the 95% connection limit are disconnected. The red arrow shows the haplotype found in the Emilia-Romagna specimens.

Molecular data indicate that parasitoids emerging from *A. spiniferus* in Emilia-Romagna belong to a distinct species of *Encarsia*, as shown by the COI gene analysis (Figure 6), with a p-distance not lower than 8.3% (Supplementary Material Table S5). The 28S gene is a less variable marker and shows that this species is more closely related to *E. smithi* (Silvestri, 1926) and *E. boswelli* (Girault, 1915) (Figure 7; p-distances ranging 1.4–2.5%; Supplementary Material Table S6). These findings strengthen the conclusion reported above to assign the species to *E. nipponica* based on morphological analysis.

## 4. Discussion

The recent identification of *Aleurocanthus spiniferus* in the Emilia-Romagna region of Italy marks a significant development in the understanding of the distribution of this invasive species in Europe. The initial detection was made through careful morphological analysis, where the distinctive spiny pupal cases and the dark adult colour with white waxy secretions were key identifiers. These characteristics are essential to distinguish *A. spiniferus* from other species of whiteflies commonly found in the region. However, given the economic importance of the agricultural sector in Emilia-Romagna, which includes extensive viticulture and fruit production, morphological identification alone was deemed insufficient.

Molecular techniques were employed to ensure accurate identification, focussing on the mitochondrial cytochrome c oxidase I (COI) gene. The sequences obtained were compared with existing GenBank entries, confirming a high similarity with *A. spiniferus* populations from other Italian regions [11]. Furthermore, a new haplotype was also found, defined here as H5, raising concerns about the potential impact of the pest on local agriculture. H5 was previously found in Chinese specimens from Yichang, Hubei province [33]. This molecular evidence is particularly important as it provides crucial insight into the potential invasion pathways of *A. spiniferus*, suggesting the possibility of a new, separate introduction event rather than a simple northward expansion from existing European populations. It is presently unclear how this haplotype has reached Italy, and, in particular, Emilia-Romagna region: its presence can be explained by a failure of its scoring in previous analyses [10,11], maybe due to its lower frequency. On the other hand, this new finding can be explained by new accidental introduction(s), probably from China, that have targeted the region specifically. Multiple introductions have previously been recorded for other invasive insects in Italy, such as for the stinkbug *H. halys* [52]. The presence of *A. spiniferus* in Emilia-Romagna could therefore be due to two non-mutually exclusive scenarios: (a) natural expansion of the populations already present in other Italian regions and/or in Albania, Croatia and Greece (as indicated by the presence of haplotypes H1 and H2); (b) newly introduced specimens from China (haplotype H5).

The impact of *A. spiniferus* on different host plants has been well documented in other regions, showing a wide host range that includes citrus, tea, and various ornamental plants. In Asia, where the pest is endemic, significant infestations of citrus crops have been reported, leading to substantial economic losses [19]. Similarly, in the newly invaded regions of southern Italy, including Calabria and Puglia, *A. spiniferus* has been found on citrus plants, grapevines and even ornamental plants, demonstrating its adaptability and potential to cause widespread damage across different plant species [11].

In Emilia-Romagna, our field survey revealed significant variation in *A. spiniferus* infestation levels among the different host plants. Specifically, the highest abundance of puparia was observed on grapevine and pear, while apple exhibited significantly lower infestation. We must explicitly state that these observations are based on a single sampling event and, therefore, do not suffice to establish a definitive host preference for the region. Consequently, multi-season studies are necessary to fully assess patterns of host plant utilisation and validate these initial findings. Nonetheless, our results are valuable as they are consistent with observations from other newly invaded European territories. For instance, Kapantaidaki et al. (2019) [9] reported high infestation levels on grapevine in Greece, while Nugnes et al. (2020) [10] documented similar trends on citrus and grapevine in southern Italy, suggesting that these plants may provide more favourable conditions for the pest’s development. The reasons for such differential infestation levels are likely multifactorial, potentially involving aspects such as plant physiology, leaf surface characteristics, and chemical cues that influence the oviposition behaviour of *A. spiniferus*. The pest’s demonstrated ability to thrive on a wide variety of host plants suggests that its spread could have far-reaching consequences for different agricultural sectors. This adaptability highlights the importance of early detection and rapid response to prevent the establishment and spread of *A. spiniferus* in new areas. Therefore, the situation in Emilia-Romagna not only represents a new challenge for local farmers but also provides a crucial case study for understanding the dynamics of this invasive pest and informing future management strategies across Europe.

A central and internationally significant finding of this study is the first documented record of *Encarsia nipponica* in Europe, specifically as a parasitoid of *A. spiniferus* in the Emilia-Romagna region of Italy. This discovery represents a major biogeographical event and establishes a new exotic host-parasitoid association for the continent, providing the essential first step for developing future sustainable management strategies. This is the third discovery of a previously unrecorded species of Aphelinidae associated with *A. spiniferus* in the country, following the discovery of *Eretmocerus iulii* (*serius* group) in 2024 [15,24], and of the congeneric *Encarsia smithi* earlier this year [53]. Being *E. iulii* first described in Italy, it is difficult to consider it to be exotic for certain. However, the same is not true for both *Encarsia* species. While these latter taxa can be promising taxa for their use in biological control of *A. spiniferus*, their impact on native fauna is presently unknown, advocating for more studies before employing them for controlling OSW.

This finding significantly expands the known geographic range of *E. nipponica*, which was previously reported mainly in Asian regions, including Japan and Taiwan [51]. This discovery is particularly important given the increasing incidence of *A. spiniferus* in Europe, especially in fruit-producing regions [13,14].

*Encarsia nipponica* has historically been placed within the *parvella* species-group (Huang and Polaszek 1998) [23]; however, based on our assessment it appears to be better placed within the *perflava* species-group. The main characteristics of the *E. perflava* group are as follows: Fore wing with an asetose area around the stigma and in the lower distal area (Figure 3D); antenna with F1 and F2 much shorter than the remaining antennomeres, and without longitudinal sensilla (Figure 3C). The wing characters are shared by the closely related *parvella* and *longifasciata* groups.

Our phylogenetic analysis suggests that *E. nipponica* is aligned with taxa currently placed in the *perflava*, *boswelli*, *lahorensis*, *smithi*, and *longifasciata* species-groups. The relationship between these species-groups is poorly understood and inconclusively supported in our phylogenetic hypothesis. Ongoing investigations in the taxonomy and phylogenetics of the *Encarsia* species groups aim to better understand the limits between these species-groups.

The parasitisation rate observed in this study was relatively low, with a parasitism impact of 0.015% on the *A. spiniferus* population. It is crucial to interpret this figure with caution, as our assessment was based on a single sampling event. However, this low rate is, in itself, an important baseline observation, suggesting that the parasitoid is either very recently established, still adapting to local environmental conditions, or facing competition with other parasitoid species. This initial rate is substantially lower than the parasitism rates reported for other *Encarsia* species used in biological control programmes. For instance, *Encarsia smithi* has achieved parasitism rates of up to 80% in controlling *A. spiniferus* in tropical regions [24]. Notably, during a weekly sampling of 200 citrus plants in Portici (Province of Naples, Campania) and a monthly survey in the eastern part of Sicily, *E. iulii* showed higher parasitism levels, reaching maximum peaks of 21.99% and 32.41%, respectively [28,29]. This suggests that *E. iulii* currently plays a dominant role in suppressing *A. spiniferus* populations in Southern Italy [54]. While our study’s objective was to confirm the parasitoid’s presence and identity rather than assess its full ecological role, this finding effectively frames the need for future research. Since our study was based on a single sampling event, more extensive and regular monitoring is needed to accurately evaluate the parasitoid community’s composition and effectiveness, especially in Northern Italy. Finally, more studies are needed to assess the long-term establishment and efficacy of *E. nipponica* in European ecosystems, as well as its potential interactions with native parasitoid species.

In conclusion, this study delivers three distinct and robust findings of international relevance: the first confirmed record of *E. nipponica* in Europe, the discovery of a new Asian haplotype of *A. spiniferus* on the continent, and a crucial baseline assessment of their initial interaction. Although the observed parasitisation rate was low, and the sampling geographically focused, this study provides a vital and timely foundation for future research and monitoring. The establishment of this parasitoid could represent an important step forward in the integrated management of this invasive pest. Ongoing monitoring and further research will be crucial in understanding the dynamics of this new parasitoid-host interaction and to optimise its use in European agroecosystems.

## Figures and Tables

**Table 1:** Information on species, sampling year, sites, and host plants for specimens utilised for molecular analyses in the present study.

Specimen Code	Preliminary Identification	Year	Sampling Site	Host Plant/Host
As GBP V1	*Aleurocanthus* sp.	2022	Giardino Botanico La Pica San Felice sul Panaro	*Vitis vinifera*
As GBP V2	*Aleurocanthus* sp.	2022	Giardino Botanico La Pica San Felice sul Panaro	*Vitis vinifera*
As GBP V3	*Aleurocanthus* sp.	2022	Giardino Botanico La Pica San Felice sul Panaro	*Vitis vinifera*
As GBP V4	*Aleurocanthus* sp.	2022	Giardino Botanico La Pica San Felice sul Panaro	*Vitis vinifera*
As GBP M1	*Aleurocanthus* sp.	2022	Giardino Botanico La Pica San Felice sul Panaro	*Pyrus communis*
As GBP M2	*Aleurocanthus* sp.	2022	Giardino Botanico La Pica San Felice sul Panaro	*Pyrus communis*
As GBP M3	*Aleurocanthus* sp.	2022	Giardino Botanico La Pica San Felice sul Panaro	*Pyrus communis*
As GBP M4	*Aleurocanthus* sp.	2022	Giardino Botanico La Pica San Felice sul Panaro	*Pyrus communis*
As M226 1	*Aleurocanthus* sp.	2022	Azienda Magnanini Carpi	*Pyrus communis*
As M226 2	*Aleurocanthus* sp.	2022	Azienda Magnanini Carpi	*Pyrus communis*
As M226 3	*Aleurocanthus* sp.	2022	Azienda Magnanini Carpi	*Pyrus communis*
As bom 5	*Aleurocanthus* sp.	2022	Bomporto	Orchard hedge
25a	*Encarsia* sp.	2022	Giardino Botanico La Pica S. Felice sul Panaro	*Aleurocanthus* sp.
25b	*Encarsia* sp.	2022	Giardino Botanico La Pica S. Felice sul Panaro	*Aleurocanthus* sp.
41a	*Encarsia* sp.	2022	Giardino Botanico La Pica S. Felice sul Panaro	*Aleurocanthus* sp.
53a	*Encarsia* sp.	2022	Giardino Botanico La Pica S. Felice sul Panaro	*Aleurocanthus* sp.
53b	*Encarsia* sp.	2022	Giardino Botanico La Pica S. Felice sul Panaro	*Aleurocanthus* sp.
56a	*Encarsia* sp.	2022	Giardino Botanico La Pica S. Felice sul Panaro	*Aleurocanthus* sp.

**Table 2:** Results from a generalised linear mixed model (GLMM) with a negative binomial distribution. The table shows the median count with interquartile range (IQR) in square brackets for each tree species (*Malus domestica*; *Pyrus communis* and *Vitis vinifera*), along with the estimated effects of each species relative to *M. domestica.* The z-value and *p*-value indicate the statistical significance of the difference in the number of *Aleurocanthus spiniferus* puparia compared to *M. domestica.*

	Median [IQR]	Estimate	*z*-Value	*p*-Value
*Malus domestica*	1 [0, 2]	-	-	-
*Pyrus communis*	4 [1, 8]	1.45	22.21	<0.001
*Vitis vinifera*	16 [8, 27]	2.891	45.88	<0.001

## Data Availability

The original contributions presented in this study are included in the article/Supplementary Material. Further inquiries can be directed to the corresponding author.

## Supplementary Materials

The following supporting information can be downloaded at: https://www.mdpi.com/article/10.3390/insects16111181/s1, Supplementary Table S1: GenBank sequences utilised for comparison. (NA: geographic origin not available); Supplementary Material Table S2: GenBank sequences used for comparison; Supplementary Material Table S3: Genetic distance values (*p*-distance) for the COI gene among *Aleurocanthus* species. The analysis was carried out on a dataset of 657 bp. Newly analysed specimens are in bold; Supplementary Material Table S4: Genetic distance values (*p*-distance) for the 16S gene among *Aleurocanthus* species. The analysis was carried out on a dataset of 488 bp. Newly analysed specimens are in bold; Supplementary Material Table S5: Genetic distance values (*p*-distance) for the COI gene among *Encarsia* species. The analysis was carried out on a dataset of 672 bp. Newly analysed specimens are in bold; Supplementary Material Table S6: Genetic distance values (*p*-distance) for the 28S gene among *Encarsia* species. The analysis was carried out on a dataset of 858 bp. Newly analysed specimens are in bold.

## References

[1] Martin J.H. (1987). An identification guide to common whitefly pest species of the world (Homoptera: Aleyrodidae). Trop. Pest Manag..

[2] EFSA Panel on Plant Health (2018). Evaluation of a paper by Guarnaccia et al. (2017) on the first report of *Phyllosticta citricarpa* in Europe. EFSA J..

[3] Van den Berg M.A., De Beer M.S. Natural enemies of the spiny blackfly, *Aleurocanthus spiniferus* (Hem.: Aleyrodidae), in Mpumalanga South Africa. Proceedings of the International Society of Citriculture.

[4] Gillespie P.S. (2012). A review of the whitefly genus *Aleurocanthus* Quaintance & Baker (Hemiptera: Aleyrodidae) in Australia. Zootaxa.

[5] EPPO. *Aleurocanthus spiniferus*. https://gd.eppo.int/taxon/ALECSN.

[6] Porcelli F. (2008). First record of *Aleurocanthus spiniferus* (Homoptera: Aleyrodidae) in Apulia, Southern Italy. EPPO Bull..

[7] Šimala M., Pintar M., Milek T.M., Markotić V. Results of a two year survey (2015–2016) of quarantine whitefly species from genus *Aleurocanthus* Quaintance & Baker 1914 on citrus in Croatia. Proceedings of the 13th Slovenian Conference on Plant Protection.

[8] Radonjić S., Hrnčić S., Malumphy C. (2014). First record of *Aleurocanthus spiniferus* (Quaintance) (Hemiptera Aleyrodidae) in Montenegro. Redia.

[9] Kapantaidaki D.E., Antonatos S., Kontodimas D., Milonas P., Papachristos D.P. (2019). Presence of the invasive whitefly *Aleurocanthus spiniferus* (Hemiptera: Aleyrodidae) in Greece. EPPO Bull..

[10] Nugnes F., Laudonia S., Jesu G., Jansen M.G.M., Bernardo U., Porcelli F. (2020). *Aleurocanthus spiniferus* (Hemiptera: Aleyrodidae) in some European countries: Diffusion, hosts, molecular characterization, and natural enemies. Insects.

[11] Streito J.C., Mendes E., Sanquer E., Strugarek M., Ouvrad D., Robin-Havret V., Poncet L., Lannou C., Rossi J.P. (2023). Incursion preparedness, citizen science and early detection of invasive insects: The case of *Aleurocanthus spiniferus* (Hemiptera, Aleyrodidae) in France. Insects.

[12] Bariselli M., Bortolotti P.P., Nannini R. (2019). Scheda 27—*Aleurocanthus spiniferus*. Schede Tecniche Per il Riconoscimento Degli Organismi Nocivi da Quarantena.

[13] Rapisarda C., Longo S. (2021). First report from Sicily (Italy) of the orange spiny whitefly, *Aleurocanthus spiniferus* (Quaintance) (Hemiptera: Aleyrodidae), and its potential risk for the Italian citrus industry. EPPO Bull..

[14] Melone G., Ascolese R., Nugnes F., Porcelli F., Rapisarda C., Farina A., Picciotti U., Garganese F., Laudonia S. (2024). An *Eretmocerus* species, parasitoid of *Aleurocanthus spiniferus*, was found in Europe: The secret savior of threatened plants. Sustainability.

[15] Gill R.J., Gerling D. (1990). The morphology of whiteflies. Whiteflies: Their Bionomics, Pest Status and Management.

[16] Mound L.A., Halsey S.H. (1978). Whitefly of the World.

[17] Cioffi M., Cornara D., Corrado I., Jansen M.G.M., Porcelli F. (2013). The status of *Aleurocanthus spiniferus* from its unwanted introduction in Italy to date. Bull. Insectology.

[18] Evans G. (2008). The Whiteflies (Hemiptera: Aleyrodidae) of the World and Their Host Plants and Natural Enemies.

[19] Silvestri F. (1927). Contribuzione alla conoscenza degli Aleurodidae (Insecta: Hemiptera) viventi su *Citrus* in Estremo Oriente e dei loro parassiti. II. Descrizione e notizie biologiche dei parassiti di Aleurodidi viventi su *Citrus*. Boll. Lab. Zool. Gen. Agrar. R. Ist. Super. Agrar. Portici.

[20] Van den Berg M.A., Hoppner G., Greenland J. (2000). An economic study of the biological control of the spiny blackfly, *Aleurocanthus spiniferus* (Hemiptera: Aleyrodidae), in a citrus orchard in Swaziland. Biocontrol Sci. Technol..

[21] Muniappan R., Purea M., Sengebau F., Reddy G.V.P. (2006). Orange spiny whitefly, *Aleurocanthus spiniferus* (Quaintance) (Homoptera: Aleyrodidae), and its parasitoids in the Republic of Palau. Proc. Hawaii. Entomol. Soc..

[22] Gyeltshen J., Hodges A., Hodges G.S. (2019). Orange Spiny Whitefly, Aleurocanthus spiniferus Quaintance (Insecta: Hemiptera: Aleyrodidae).

[23] Huang J., Polaszek A. (1998). A revision of the Chinese species of *Encarsia* Förster (Hymenoptera: Aphelinidae): Parasitoids of whiteflies, scale insects and aphids (Hemiptera: Aleyrodidae, Diaspididae, Aphidoidea). J. Nat. Hist..

[24] Myartseva S.N. (2007). Species of genus *Encarsia* Förster (Hymenoptera: Aphelinidae) parasitoids of whiteflies (Hemiptera: Aleyrodidae) associated with *Psidium guajava* L. in Mexico, with key and description of new species. Biosystematica.

[25] Shih Y.T., Ko C.C. (2020). Taiwan Insect Fauna Aphelinidae.

[26] Colazza S., Bin F. (1995). Efficiency of *Trissolcus basalis* (Hymenoptera: Scelionidae) as an egg parasitoid of *Nezara viridula* (Heteroptera: Pentatomidae) in Central Italy. Environ. Entomol..

[27] (1992). Council Directive 92/43/EEC of 21 May 1992 on the conservation of natural habitats and of wild fauna and flora. Off. J. Eur. Union.

[28] Laudonia S., Melone G., Ascolese R., Nugnes F. (2024). *Eretmocerus iulii* Laudonia et Melone sp. n.: Parasitoid associated with *Aleurocanthus spiniferus*. Bull. Insectology.

[29] Noyes J.S. (1982). Collecting and preserving chalcid wasps (Hymenoptera: Chalcidoidea). J. Nat. Hist..

[30] Brooks M.E., Kristensen K., van Benthem K.J., Magnusson A., Berg C.W., Nielsen A., Skaug H.J., Mächler M., Bolker B.M. (2017). glmmTMB balances speed and flexibility among packages for zero-inflated generalized linear mixed modeling. R J..

[31] R Core Team (2020). R: A Language and Environment for Statistical Computing.

[32] Nugnes F., Russo E., Viggiani G., Bernardo U. (2018). First record of an invasive fruit fly belonging to *Bactrocera dorsalis* complex (Diptera: Tephritidae) in Europe. Insects.

[33] Uesugi R., Sato Y., Han B., Huang Z.D., Yara K., Furuhashi K. (2016). Molecular evidence for multiple phylogenetic groups within two species of invasive spiny whiteflies and their parasitoid wasp. Bull. Entomol. Res..

[34] Simon C., Frati F., Beckenbach A., Crespi B., Liu H., Flook P. (1994). Evolution, weighting, and phylogenetic utility of mitochondrial gene sequences and a compilation of conserved Polymerase Chain Reaction primers. Ann. Entomol. Soc. Am..

[35] Folmer O., Black M., Hoeh W., Lutz R., Vrijenhoek R. (1994). DNA primers for amplification of mitochondrial cytochrome c oxidase subunit I from diverse metazoan invertebrates. Mol. Mar. Biol. Biotechnol..

[36] Nunn G.B., Theisen B.F., Christensen B., Arctander P. (1997). Simplicity correlated size growth of the nuclear 28S ribosomal RNA D3 expansion segment in the crustacean order Isopoda. J. Mol. Evol..

[37] Polaszek A., Ayshford T., Yahya B.E., Fusu L. (2014). *Wallaceaphytis*: An unusual new genus of parasitoid wasp (Hymenoptera: Aphelinidae) from Borneo. J. Nat. Hist..

[38] Kumar D., Singh M., Kushwaha M., Makarana G., Yadav M.R. (2021). Integrated use of organic and inorganic nutrient sources influences the nutrient content, uptake and nutrient use efficiencies of fodder oats (*Avena sativa*). Indian J. Agron..

[39] Xia X. (2018). DAMBE7: New and improved tools for data analysis in molecular biology and evolution. Mol. Biol. Evol..

[40] Templeton A.R., Crandall K.A., Sing C.F. (1992). A cladistic analysis of phenotypic associations with haplotypes inferred from restriction endonuclease mapping and DNA sequence data. III. Cladogram estimation. Genetics.

[41] Clement M., Posada D., Crandall K.A. (2000). TCS: A computer program to estimate gene genealogies. Mol. Ecol..

[42] Múrias dos Santos A., Cabezas M.P., Tavares A.I., Xavier R., Branco M. (2016). tcsBU: A tool to extend TCS network layout and visualization. Bioinformatics.

[43] Katoh K., Standley D.M. (2013). MAFFT multiple sequence alignment software version 7: Improvements in performance and usability. Mol. Biol. Evol..

[44] Borowiec M.L. (2019). Spruceup: Fast and flexible identification, visualization, and removal of outliers from large multiple sequence alignments. J. Open-Source Softw..

[45] Minh B.Q., Schmidt H.A., Chernomor O., Schrempf D., Woodhams M.D., von Haeseler A., Lanfear R. (2020). IQ-TREE 2: New models and efficient methods for phylogenetic inference in the genomic era. Mol. Biol. Evol..

[46] Hoang D.T., Chernomor O., von Haeseler A., Minh B.Q., Vinh L.S. (2018). UFBoot2: Improving the Ultrafast Bootstrap Approximation. Mol. Biol. Evol..

[47] Martin J.H. (1999). The Whitefly Fauna of Australia (Sternorrhyncha: Aleyrodidae): A Taxonomic Account and Identification Guide.

[48] Dubey A.K., Sundararaj R. (2005). Whitefly species of the genus *Aleurocanthus* Quaintance & Baker (Hemiptera: Aleyrodidae) from India, with descriptions of six new species. Orient. Insects.

[49] Dubey A.K., Ko C.C. (2012). Sexual dimorphism among species of *Aleurocanthus* Quaintance & Baker (Hemiptera: Aleyrodidae) in Taiwan, with one new species and an identification key. Zootaxa.

[50] Jansen M., Porcelli F. (2018). *Aleurocanthus camelliae* (Hemiptera: Aleyrodidae), a species possibly new for the European fauna of a genus in great need of revision. Tijdschr. Entomol..

[51] Viggiani G. (1987). Le specie italiane del genere *Encarsia* Foerster (Hymenoptera: Aphelinidae). Boll. Lab. Entomol. Agrar. Filippo Silvestri Portici.

[52] Cesari M., Maistrello L., Piemontese L., Bonini R., Dioli P., Lee W., Park C.G., Partsinevelos G.K., Rebecchi L., Guidetti R. (2018). Genetic diversity of the brown marmorated stink bug *Halyomorpha halys* in the invaded territories of Europe and its patterns of diffusion in Italy. Biol. Invasions.

[53] Melone G., Andretta L., Pica F., Donnarumma F.P., Ascolese R., Nugnes F., Laudonia S. (2025). First Detection of *Encarsia smithi* in Italy and co-occurrence with *Eretmocerus iulii*: A case of unintentional introductions and new associations with the invasive species *Aleurocanthus spiniferus*. Insects.

[54] Farina A., Rapisarda C. (2025). Parasitization Activity by *Eretmocerus iulii* over the Orange Spiny Whitefly, *Aleurocanthus spiniferus*, in Sicily. Insects.

