# Supplementary material for: A European Début: The Asian Parasitoid Encarsia nipponica Targets the Invasive Aleurocanthus spiniferus in Northern Italy
Source: Insects. 2025 Nov 19;16(11):1181. doi: 10.3390/insects16111181 (PMC12653755; doi:10.3390/insects16111181)
Supplement: Supplementary file 1 [file insects-16-01181-s001.zip › Table S1.pdf]

Supplementary Material Table S1. GenBank sequences utilized for comparison. (NA: geographic origin not available)

Species	GenBank Accession Number	Country
**Whitefly COI**		
Aleurocanthus camelliae	KU761949	NA
Aleurocanthus spiniferus	NC_029155	China
Aleurocanthus spiniferus	MW183549	China
Aleurocanthus spiniferus	MN662884	Italy
Aleurocanthus spiniferus	MN662885	Italy
Aleurocanthus spiniferus	MN662886	Italy
Aleurocanthus spiniferus	MN662887	Italy
Aleurocanthus spiniferus	MN662888	Italy
Aleurocanthus spiniferus	MN662889	Italy
Aleurocanthus spiniferus	MN662893	Italy
Aleurocanthus spiniferus	MN662894	Italy
Aleurocanthus spiniferus	MN662895	Italy
Aleurocanthus spiniferus	MN662896	Italy
Aleurocanthus spiniferus	MN662897	Italy
Aleurocanthus spiniferus	MN662898	Italy
Aleurocanthus spiniferus	MN662899	Italy
Aleurocanthus spiniferus	MN662900	Italy
Aleurocanthus spiniferus	MN662901	Italy
Aleurocanthus spiniferus	MN662902	Italy
Aleurocanthus spiniferus	MN662912	Italy
Aleurocanthus spiniferus	MN662913	Italy
Aleurocanthus spiniferus	MN662914	Italy
Aleurocanthus spiniferus	MN662915	Italy
Aleurocanthus spiniferus	MN662916	Italy
Aleurocanthus spiniferus	MN662917	Italy
Aleurocanthus spiniferus	MN662918	Italy
Aleurocanthus spiniferus	MN662919	Italy
Aleurocanthus spiniferus	MH700443	Greece
Aleurocanthus spiniferus	MN662890	Italy
Aleurocanthus spiniferus	MN662891	Italy
Aleurocanthus spiniferus	MN662892	Italy
Aleurocanthus spiniferus	MN662903	Italy
Aleurocanthus spiniferus	MN662904	Italy
Aleurocanthus spiniferus	MN662905	Italy
Aleurocanthus spiniferus	MN662906	Italy
Aleurocanthus spiniferus	MN662907	Italy
Aleurocanthus spiniferus	MN662908	Italy
Aleurocanthus spiniferus	MN662909	Albania
Aleurocanthus spiniferus	MN662910	Albania
Aleurocanthus spiniferus	MN662911	Albania
Aleurocanthus spiniferus	MN662920	Italy

Aleurocanthus spiniferus	MN662921	Italy
Aleurocanthus spiniferus	MN662922	Italy
Aleurocanthus spiniferus	MN662923	Albania
Aleurocanthus spiniferus	MN662924	Albania
Aleurocanthus spiniferus	MN662925	Italy
Aleurocanthus spiniferus	MH700444	Greece, Italy
Aleurocanthus spiniferus	AB786720	China
Aleurocanthus spiniferus	MH700445	Greece, Italy
Aleurocanthus spiniferus	MH700446	Greece
Aleurocanthus spiniferus	AB786715	China
Aleurocanthus spiniferus	AB786716	China
Aleurocanthus spiniferus	AB786717	China
Aleurocanthus spiniferus	AB786718	China
Aleurocanthus spiniferus	AB786719	China
Aleurocanthus spiniferus	AB786721	China
Aleurocanthus spiniferus	AB786723	China
Aleurocanthus spiniferus	AB786722	China
Aleurocanthus spiniferus	AB615363	Japan
Aleurocanthus spiniferus	AB615364	Japan
Aleurocanthus spiniferus	AB536792	Japan
Aleurocanthus spiniferus	AB558172	Japan
Aleurocanthus spiniferus	AB536793	Japan
Aleurocanthus spiniferus	OR495607	Croatia
Aleurocanthus spiniferus	OR493470	Croatia
Aleurocanthus spiniferus	OR493434	Croatia
Aleurocanthus spiniferus	OR499882	Croatia

Whitefly 16S

Aleurocanthus woglumi	JX281761	NA
Aleurocanthus camelliae	KU761949	NA
Aleurocanthus spiniferus	KJ437166	NA
Aleurocanthus spiniferus	OQ180915	Japan

Parasitoid COI

Aphelinus abdominalis	PP480258	United Kingdom
Encarsia guadeloupae	KY607910	India
Encarsia guadeloupae	MT026001	NA
Encarsia lounsburyi	MT894183	Ethiopia
Encarsia lounsburyi	MT894184	Ethiopia
Encarsia lounsburyi	MT894185	Ethiopia
Encarsia lounsburyi	MH456759	Chile
Encarsia lounsburyi	MH456760	Chile
Encarsia lounsburyi	MH456761	Chile
Encarsia lounsburyi	MH456762	Chile
Encarsia lounsburyi	MH456763	Chile
Encarsia perniciosi	MN057949	Australia

Encarsia perniciosi	MN057950	Australia
Encarsia perniciosi	KX065213	France
Encarsia perniciosi	MH460407	U.S.A.
Encarsia cf. *perniciosi*	KX065211	France
Encarsia citrina	MT894186	Ethiopia
Encarsia citrina	MT894187	Ethiopia
Encarsia citrina	MN057951	Australia
Encarsia citrina	MN057952	Australia
Encarsia citrina	MN065499	Australia
Encarsia citrina	MH456482	Chile
Encarsia citrina	MH928406	Indonesia
Encarsia citrina	MH928635	Indonesia
Encarsia citrina	MH928822	Indonesia
Encarsia citrina	MF444685	NA
Encarsia citrina	KF778395	U.S.A.
Encarsia citrina	KF778398	U.S.A.
Encarsia citrina	KF778399	U.S.A.
Encarsia citrina	KF778403	U.S.A.
Encarsia citrina	KF778404	U.S.A.
Encarsia citrina	KF778405	U.S.A.
Encarsia citrina	KF778406	U.S.A.
Encarsia citrina	KF778407	U.S.A.
Encarsia citrina	KF778408	U.S.A.
Encarsia citrina	KF778418	Japan
Encarsia citrina	KF778419	Japan
Encarsia citrina	KF778435	Japan
Encarsia citrina	KF778441	Japan
Encarsia citrina	KF778446	NA
Encarsia citrina	KF778452	Japan
Encarsia citrina	KF778462	Japan
Encarsia citrina	KF778463	Japan
Encarsia citrina	KF778465	Japan
Encarsia citrina	KF778478	Japan
Encarsia citrina	KF778479	Japan
Encarsia citrina	KF778480	Japan
Encarsia citrina	KF778482	Japan
Encarsia citrina	KF778483	Japan
Encarsia citrina	KF778485	Japan
Encarsia citrina	KF778486	Japan
Encarsia citrina	KF778488	Japan
Encarsia citrina	KF778490	U.S.A.
Encarsia citrina	KF778491	U.S.A.
Encarsia citrina	KF778493	U.S.A.
Encarsia citrina	KF778492	U.S.A.
Encarsia citrina	KF778494	U.S.A.
Encarsia citrina	KF778495	U.S.A.
Encarsia citrina	KF778496	U.S.A.
Encarsia citrina	KF778497	U.S.A.

Encarsia citrina	KF778498	U.S.A.
Encarsia citrina	KF778499	U.S.A.
Encarsia citrina	KF778500	U.S.A.
Encarsia citrina	KF778501	U.S.A.
Encarsia citrina	KF778502	U.S.A.
Encarsia citrina	KF778503	U.S.A.
Encarsia citrina	KF778504	U.S.A.
Encarsia citrina	KF778505	U.S.A.
Encarsia citrina	KF778506	U.S.A.
Encarsia citrina	KF778507	U.S.A.
Encarsia citrina	KF778508	U.S.A.
Encarsia citrina	KF778509	U.S.A.
Encarsia citrina	KF778510	Japan
Encarsia citrina	KF778511	Japan
Encarsia citrina	KF778513	Japan
Encarsia citrina	KF778514	Japan
Encarsia citrina	KF778515	Japan
Encarsia citrina	KF778516	Japan
Encarsia hispida	MH456571	Chile
Encarsia hispida	MH456572	Chile
Encarsia hispida	MH456573	Chile
Encarsia hispida	MH456574	Chile
Encarsia longifasciata	MH926950	Indonesia
Encarsia herndoni	MH926974	Indonesia
Encarsia cibcensis	MH927153	Indonesia
Encarsia gracilens	MH928979	Indonesia
Encarsia inquirenda	MH928989	Indonesia
Encarsia inquirenda	KX065212	France
Encarsia inquirenda	JQ268914	Iran
Encarsia perplexa	MF444686	NA
Encarsia tamaulipeca	MF444687	NA
Encarsia inaron	KY833503	Pakistan
Encarsia inaron	KY839153	Pakistan
Encarsia inaron	KY839723	Pakistan
Encarsia inaron	KF055389	Iran
Encarsia inaron	KF055390	Iran
Encarsia inaron	KF055391	Iran
Encarsia inaron	KF055392	Iran
Encarsia inaron	KF055393	Iran
Encarsia inaron	KF055394	Iran
Encarsia inaron	KF055395	Iran
Encarsia inaron	KF055396	Iran
Encarsia inaron	GQ423483	U.S.A.
Encarsia inaron	GQ423484	Italy
Encarsia vandrieschei	KF778400	Japan
Encarsia vandrieschei	KF778401	Japan
Encarsia vandrieschei	KF778512	Japan
Encarsia normarki	KF778411	Japan

Encarsia normarki	KF778415	Japan
Encarsia normarki	KF778416	Japan
Encarsia normarki	KF778453	Japan
Encarsia normarki	KF778459	Japan
Encarsia normarki	KF778484	Japan
Encarsia brimblecombei	KF778433	Japan
Encarsia brimblecombei	KF778437	Japan
Encarsia brimblecombei	KF778440	Japan
Encarsia brimblecombei	KF778458	Japan
Encarsia brimblecombei	KF778461	Japan
Encarsia schmidti	KF778431	Japan
Encarsia schmidti	KF778464	Japan
Encarsia schmidti	KF778475	Japan
Encarsia schmidti	KF778476	Japan
Encarsia schmidti	KF778477	Japan
Encarsia iris	HQ660515	NA
Encarsia iris	JF750716	Australia
Encarsia iris	JF750717	Australia
Encarsia iris	JF750718	Australia
Encarsia iris	JF750719	Australia
Encarsia diaspidicola	GQ922196	Samoa
Encarsia diaspidicola	GQ922197	Samoa
Encarsia diaspidicola	GQ922198	Samoa
Encarsia berlesei	KT884744	Iran
Encarsia berlesei	GQ922199	U.S.A.
Encarsia berlesei	GQ922200	U.S.A.
Encarsia berlesei	GQ922201	U.S.A.
Encarsia hera	OP270223	U.S.A.
Encarsia hera	OP270224	U.S.A.
Encarsia formosa	MG441298	Canada
Encarsia formosa	FM210158	NA
Encarsia formosa	FM210159	NA
Encarsia formosa	FM210160	NA
Encarsia formosa	MG813797	NA

Parasitoid 28S

Encarsia formosa	MG727856	NA
Encarsia formosa	MG596962	NA
Encarsia smithi	AF254233	NA
Encarsia smithi	AF254234	NA
Encarsia smithi	LC088727	Japan
Encarsia smithi	LC088728	Japan
Encarsia smithi	LC088729	Japan
Encarsia smithi	LC088730	Japan
Encarsia aphania	OQ683545	Costa Rica
Encarsia aphania	OQ683546	Belize
Encarsia avida	OQ683547	Costa Rica

Encarsia catula	OQ683548	Costa Rica
Encarsia dichaeta	OQ683549	Costa Rica
Encarsia dichaeta	OQ683550	Costa Rica
Encarsia dichaeta	OQ683551	Costa Rica
Encarsia dichaeta	OQ683552	Costa Rica
Encarsia inbioa	OQ683553	Costa Rica
Encarsia acusa	OQ683554	Costa Rica
Encarsia gr. *mexicana*	OQ683555	Ecuador
Encarsia gr. *mexicana*	OQ683556	Costa Rica
Encarsia venia	OQ683557	Costa Rica
Encarsia svetlana	OQ683558	Guyana
Encarsia fredbennetti	OQ683559	Trinidad and Tobago
Encarsia mexicana	OQ683560	Costa Rica
Encarsia noora	OQ683561	Costa Rica
Encarsia noora	OQ683562	Costa Rica
Encarsia marynoyesae	OQ683563	Costa Rica
Encarsia tamaulipeca	OQ683564	Ecuador
Encarsia gr. noyesi	OQ683565	Ecuador
Encarsia gr. *noyesi*	OQ683566	Costa Rica
Encarsia cubensis	OQ683567	Costa Rica
Encarsia lounsburyi	OQ683568	Costa Rica
Encarsia citrina	OQ683569	United Kingdom
Encarsia boswelli	OQ683570	India
Encarsia opulenta	OQ683571	Mexico
Encarsia protransvena	PQ451935	Italy
